# Characterisation of Flavour Attributes in Egg White Protein Using HS-GC-IMS Combined with E-Nose and E-Tongue: Effect of High-Voltage Cold Plasma Treatment Time

**DOI:** 10.3390/molecules27030601

**Published:** 2022-01-18

**Authors:** Mustapha Muhammad Nasiru, Muhammad Umair, Evans Frimpong Boateng, Fawze Alnadari, Kashif-ur Rehman Khan, Zhaobin Wang, Ji Luo, Wenjing Yan, Hong Zhuang, Ali Majrashi, Jianhao Zhang, Sameh A. Korma

**Affiliations:** 1 National Centre of Meat Quality and Safety Control, Collaborative Innovation Centre of Meat Production and Processing, Quality and Safety Control, College of Food Science and Technology, Nanjing Agricultural University, Nanjing 210095, China; 2018208036@njau.edu.cn (M.M.N.); nanafrimpong94@yahoo.com (E.F.B.); 2017208001@njau.edu.cn (Z.W.); ywj1103@njau.edu.cn (W.Y.); 2 College of Food Science and Technology, Nanjing Agricultural University, Nanjing 210095, China; 2019208038@njau.edu.cn; 3 Department of Food Science and Technology, Faculty of Renewable Natural Resources, Federal University Dutsin-Ma, Katsina 821221, Nigeria; 4 Department of Food Science and Engineering, College of Chemistry and Environmental Engineering, Shenzhen University, Shenzhen 518060, China; umair_uaf@hotmail.com; 5 Department of Pharmaceutical Chemistry, Faculty of Pharmacy, The Islamia University of Bahawalpur, Bahawalpur 63100, Pakistan; kashifur.rahman@iub.edu.pk; 6 College of Life Science, Anhui Normal University, Wuhu 241000, China; luoji2019336@ahnu.edu.cn; 7 Quality and Safety Assessment Research Unit, U.S. National Poultry Research Center, USDA-ARS, 950 College Station Road, Athens, GA 30605, USA; hong.zhuang@ars.usda.gov; 8 Department of Biology, College of Science, Taif University, P.O. Box 11099, Taif 21944, Saudi Arabia; 9 Department of Food Science, Faculty of Agriculture, Zagazig University, Zagazig 44519, Egypt; sameh.hosny@zu.edu.eg; 10 School of Food Science and Engineering, South China University of Technology, Guangzhou 510641, China

**Keywords:** high-voltage cold plasma, egg white protein, volatile compounds, flavour, E-nose, E-tongue, PCA, PLS-DA

## Abstract

Egg white protein (EWP) is susceptible to denaturation and coagulation when exposed to high temperatures, adversely affecting its flavour, thereby influencing consumers’ decisions. Here, we employ high-voltage cold plasma (HVCP) as a novel nonthermal technique to investigate its influence on the EWP’s flavour attributes using E-nose, E-tongue, and headspace gas-chromatography-ion-mobilisation spectrometry (HS-GC-IMS) due to their rapidness and high sensitivity in identifying flavour fingerprints in foods. The EWP was investigated at 0, 60, 120, 180, 240, and 300 s of HVCP treatment time. The results revealed that HVCP significantly influences the odour and taste attributes of the EWP across all treatments, with a more significant influence at 60 and 120 s of HVCP treatment. Principal component analyses of the E-nose and E-tongue clearly distinguish the odour and taste sensors’ responses. The HS-GC-IMS analysis identified 65 volatile compounds across the treatments. The volatile compounds’ concentrations increased as the HVCP treatment time was increased from 0 to 300 s. The significant compounds contributing to EWP characterisation include heptanal, ethylbenzene, ethanol, acetic acid, nonanal, heptacosane, 5-octadecanal, decanal, p-xylene, and octanal. Thus, this study shows that HVCP could be utilised to modify and improve the EWP flavour attributes.

## 1. Introduction

Egg white protein (EWP) is an affordable source of high-quality protein containing several amino acids higher than other sources of protein, such as soybean and milk proteins [1,2,3,4]. It is highly retained in our body and has excellent physical and nutritional properties. Therefore, it is an important ingredient in various foods such as baked products, meat products, noodles, and meringues [5,6,7]. Additionally, in the course of processing and storage, EWP is regularly prone to oxidation and denaturation that results in adverse changes in its constituents and properties [1,8,9].

Flavour attributes of EWP; aroma and taste, to be precise, are the essential attributes that dictate and affect the overall flavour characteristics of the EWP, as well as determine its acceptability to consumers [1,10,11]. However, conventional food processing techniques such as thermal processes have been extensively employed in the processing of EWP, but undesirably affect the structural, functional, and nutritional attributes of the EWP, thereby affecting its distinctive flavour, causing chemical pollution, which in turn could harm consumers [8,10,12,13,14].

The nonthermal processing technique is a food processing technique that is achieved at room temperatures; it preserves food materials’ attributes whilst minimally affecting those attributes, thus preventing flavour loss, maintaining nutritional components, and extending the shelf life of the food materials [15,16]. High-voltage cold plasma (HVCP) is among the novel nonthermal processing techniques gaining attention lately from the scientific community. HVCP is comprise of ionised and un-ionised gas produced at either atmospheric or low-pressure environment; the gas contains several active species, namely electrons, free radicals, ions, neutral molecules, reactive oxygen, and nitrogen species, and charged particles [16,17,18]. Food materials subjected to HVCP retain and improve their sensitive quality attributes such as nutrients and flavour, it is also harmless to consumers as it did not leave any toxic chemicals on the HVCP-treated foods [16,19,20].

Consequently, some researchers have applied nonthermal processing techniques on EWP, namely, high-pressure processing [21,22], irradiation [13,23,24], ultrasound [25,26], and pulsed electric field [9,27,28]. Evidently, there is no study that employed HVCP on EWP. The instrumental analytical analysis technique is widely employed to characterise food flavour because it has numerous advantages (it is rapid, reliable, efficient, it has high selectivity and activity, and it can discriminate at a molecular level) over sensory analysis technique [10,29,30]. Moreover, the synergy of distinct analysis techniques provides comprehensive, consistent, and precise data on foods’ flavour [10,31]. Gas chromatography spectrometry has been used extensively to discriminate aroma components of various egg proteins, such as GC-MS for an egg yolk [32], SPME-GC-MS for egg yolk [33], HS-SPME GC-MS for egg white, and GC-O/GC-MS for egg yolk [34] proteins. GC-MS has an excellent discrimination capability, but it needs a high-vacuum working setting which restricts its mobility, additionally, it’s time consuming because it needs a lot of continual steps which reduces sample characterisation efficacy and hinders rapid identification of compounds [35]. GC-IMS however is an inexpensive and easy to operate device that uses gas phase separation and detection techniques by integrating the discrimination capability of GC with its fast, highly sensitive detection technique [36]. IMS is an analytical method for identifying volatile and semi-volatile compounds that uses a low electric field to separate the generated ions in the gas phase at atmospheric pressure [35]. Likewise, electronic nose (E-nose) has also been used to assess foods’ aroma and other attributes due to its being inexpensive and non-destructive [37]. Yongwei, et al. [38], Dutta, et al. [39], and Yimenu, et al. [40] use E-nose to assess egg quality, while Wang, et al. [41] evaluates volatile compounds of seven egg species. Furthermore, electronic tongue (E-tongue) is a collection of versatile chemical sensors with cross sensitivity utilised for classification, quantification, and prediction of flavour in foods [42,43,44,45].

The combining of several approaches reveals the more complete, dependable, and new findings of food flavour. Nevertheless, to the best of our knowledge, flavour changes during egg processing using a combination of E-nose, E-tongue, and HS-GC–IMS is seldom documented. Thus, the objectives of this study were to investigate synergetic utilisation of HS-GC-IMS, E-nose and E-tongue to characterise the flavour attributes of egg white protein using novel HVCP at various treatment times, also, to ascertain the effect of HVCP on the EWP.

## 2. Results and Discussions

### 2.1. Chemical Components Composition of High-Voltage Cold Plasma Treated Egg White Protein

The chemical composition of the HVCP-treated EWP is presented in Table 1. The results shows that there was no difference in the moisture, protein and reducing sugars’ content due to HVCP treatments. However, a significant difference (*p* ≤ 0.05) was found between the 0, 60, 120 and 180 s and 240 and 300 s of HVCP treatment for the minerals’ content. Our results corroborated with the findings of Chen, et al. [46] for freeze-dried and spray-dried egg white protein hydrolysates.

### 2.2. Analysis of Odour Attributes Using Electronic Nose

**Figure 1:**
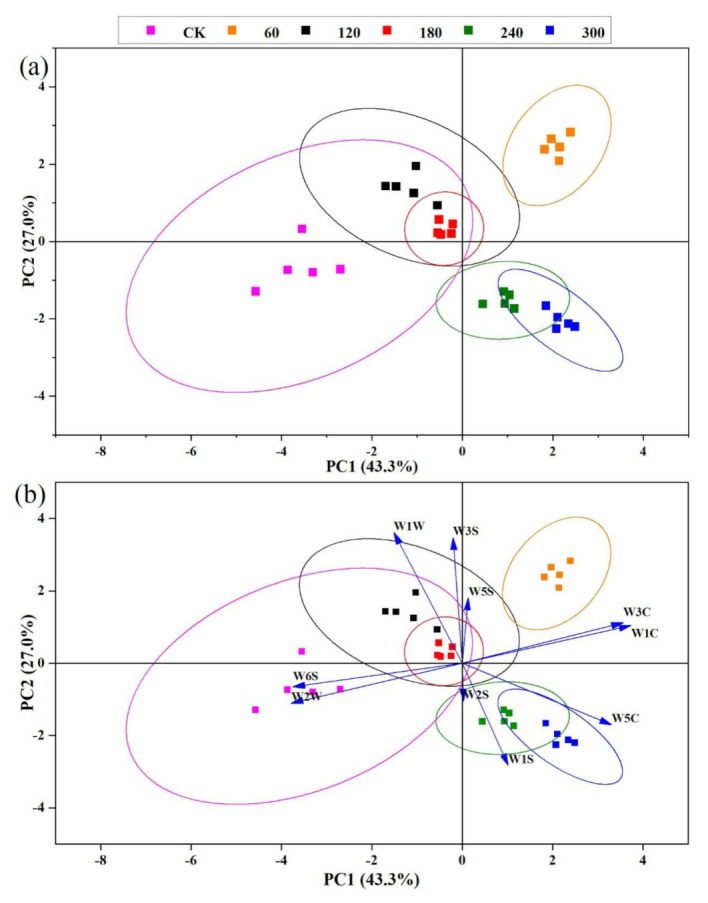
(**a**) PCA plot describing the variation in the odour fingerprints of HVCP treated EWP; (**b**) Loading plot describing the distribution of odour fingerprints across various HVCP treatments on the EWP; CK: Control; PC1: Principal component 1; PC2: Principal component 2; W1C: Aromatic compounds; W5S: Ammonia and aromatic molecules; W3C: Broad-nitrogen oxide; W6S: Hydrogen; W5C: Methane, propane and aliphatics; W1S: Broad-methane; W1W: Sulphur-containing organics; W2S: Broad-alcohols, broad-carbon chains; W2W: Aromatic, sulphur-and chlorine-containing organics and W3S: Methane and aliphatics.

Principal component analysis (PCA) is a multivariate statistical analysis technique that is primarily employed to decrease the dimensionality of a collection of data while keeping as much information as possible by excluding the variables with the lowest ranking [47]. It also can assess the consistency and variations among samples. Moreover, it is a simple, rapid, and linear statistical approach [48]. This study employed PCA and radar charts to assess the responses to discriminate flavour attributes among the HVCP treated EWP samples (Figure 1). In Figure 1a the PCA plot describes 70.3% of the EWP data from PC1 (43.3%) and PC2 (27.0%). It shows good discrimination between the control and HVCP-treated group that sufficiently explains the result. PC1 discriminates among the various HVCP-treated samples, while PC2 describes the variation between the control and HVCP-treated groups. Moreover, the 240 and 300 s, as well as 120 and 180 s of HVCP treatment, had similar odour fingerprints, while the 60 s treated group had a distinctive odour fingerprint from the other treated samples. This indicated that the odour fingerprints were higher in the HVCP-treated group than in the control and the variation among the HVCP-treated groups was described better. The W5C, W2S, and W1S sensors have positive loadings on the PC1 (Figure 1b). The HVCP treatment of 240 and 300 s could be associated with these sensors that respond to hydrocarbons, ethanol, nitrogen oxides, and methane compounds, while W3S, W5C, and W1C sensors also have positive loadings on PC2 that responds to aromatic compounds which could be due to either 60 or 300 s of HVCP treatment on EWP (Figure 1b). Conversely, W6S and W2W sensors associated with aromatic hydrocarbons and hydrogen compounds have negative loadings on PC1 that resulted from HVCP treatment of 60 s. Whereas, W1W and W3S sensors that react to sulphur and methane compounds have negative loadings on PC2 which could be attributed to either 120 or 180 s of HVCP treatment on EWP (Figure 1b).

Moreover, the result shows that HVCP treatment significantly influences the odour of the EWP. The various sensitivities of the control and treated EWP to the E-Nose sensors are presented in a radar chart (Figure 1a). Harlina et al. [49] sufficiently described E-nose data for salted duck eggs, Wang, Jin, Jin, Ma, Wang, Liu and He [41] _ENREF_47 for avian eggs, Yongwei, et al. [38] for chicken eggs, and Sun et al. [50] for egg white using PCA. No significant changes were found in the control and HVCP-treated samples from the radar chart for W6S, W5C, W1W, and W2S sensors’ values. Additionally, the W2S and W1S sensors’ values increased with increased HVCP treatment time, W1C, W3C and W5C sensors’ values decreased with increased HVCP treatment time, and no definite trend was found in W3S sensor values across the HVCP treatments. The HVCP treatment increases the availability of odour fingerprints of the EWP.

### 2.3. Analysis of Taste Attributes Using E-Tongue

**Figure 2:**
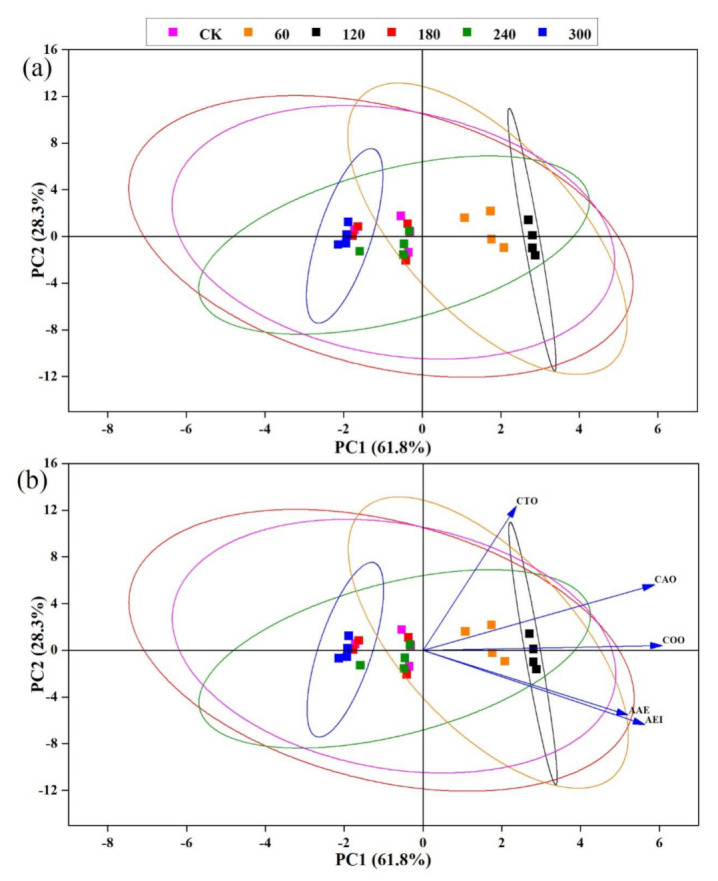
(**a**) PCA plot describing the variation in the taste fingerprints of HVCP-treated EWP; (**b**) Loading plot describing the distribution of taste fingerprints across various HVCP treatment on the EWP.CK: Control; PC1: Principal component 1; PC2: Principal component 2; AAE: Umami; CTO: Saltiness; CAO: Sourness; COO: Bitterness and AEI: Astringency.

Five tastes, namely umami (AAE), saltiness (CTO), sourness (CAO), bitterness (COO), and astringency (AEI), were evaluated from the E-tongue sensors’ response against different HVCP treatments on the EWP. From the PCA results of the taste attributes (Figure 2a), from the plot the data were divided into four clusters. The first and second clusters fell under the PC2 that described 28.3% of the data comprised of the control, 180, 240 and 300 s of HVCP treatment. However, overlapping in the above results indicates that some components of 0 (control), 180, 240 and 300 s of HVCP treatment have similar taste fingerprints. In contrast, the remaining components of the control, 60 and 120 s, overlap each other. This shows that they have similar taste attributes. Whereas 61.8% of the third and fourth clusters were sufficiently described by the PC1, which indicated that 60 and 120 s of the HVCP-treated EWP were distinctively different from each other with closely similar taste fingerprints. Additionally, from the PCA bi-plot result (Figure 2b), it could be observed that HVCP treatment for 60 and 120 s on the EWP yields excellent amounts of taste attributes as all the sensors’ responses have positive loadings on the principal component plot in contrast to the other HVCP treatments that have negative loadings.

**Figure 3:**
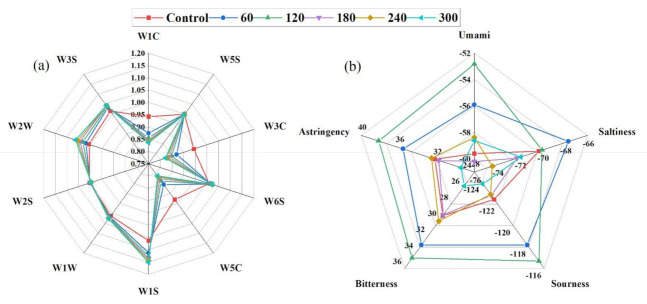
(**a**) Radar plot describing the influence of HVCP treatment on the odour fingerprints of the EWP; (**b**) Radar plot describing the influence of HVCP treatment on the taste fingerprints of the EWP.W1C: Aromatic compounds; W5S: Ammonia and aromatic molecules; W3C: Broad-nitrogen oxide; W6S: Hydrogen; W5C: Methane, propane and aliphatics; W1S: Broad-methane; W1W: Sulphur-containing organics; W2S: Broad-alcohols, broad-carbon chains; W2W: Aromatic, sulphur-and chlorine-containing organics and W3S: Methane and aliphatics. AAE: Umami; CTO: Saltiness; CAO: Sourness; COO: Bitterness and AEI: Astringency.

Conversely, Figure 3b present the E-tongue results in a radar plot. No significant differences were found in the astringency, bitterness and sourness of the EWP among the 0 (control), 180 and 240 s of HVCP treatments. Control and 120 s, as well as 180 and 300 s of HVCP treatment, showed no difference in the saltiness values of the EWP. Also, 240 and 300 s of HVCP treatment showed no significant difference in the umami values of the EWP. Furthermore, significant differences (*p* ≤ 0.05) were evident for 60 and 120 s of HVCP treatment in the umami, astringency, bitterness and sourness of the EWP; they also had the highest values of sensors’ response across the HVCP treatments and tastes parameters. However, sensor response values decreased as HVCP treatment time increased from 180 to 300 s across all taste attributes. The radar chart results corroborated with the PCA result above. A combination of PCA and radar plots were utilised to satisfactorily describe the E-tongue data in shiitake mushrooms’ flavour [51] and hen eggs’ quality assessment [52].

### 2.4. Volatile Organic Compounds Analysis

#### 2.4.1. Identification of Volatile Compounds from Plasma-Treated Egg White Protein

**Figure 4:**
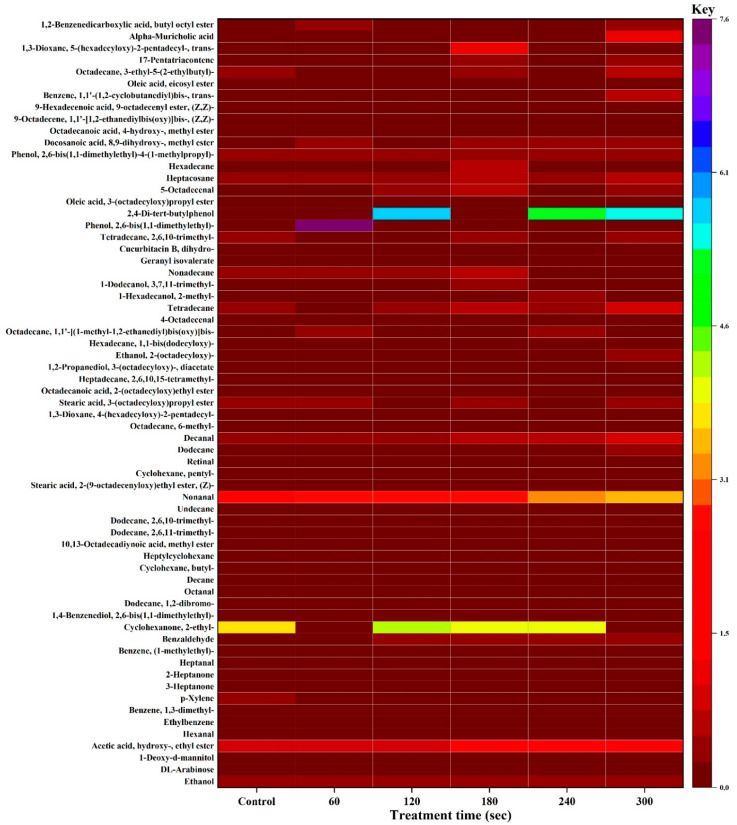
Heatmap showing individual concentration of volatile compounds identified in the HVCP-treated EWP at different treatment times.

**Figure 5:**
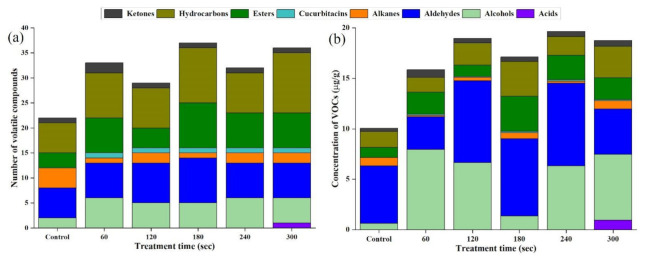
(**a**) The number of volatile compounds identified in the HVCP-treated EWP using HS-GC-IMS. (**b**) The concentrations of volatile compounds classes identified in the HVCP treated EWP using HS-GC-IMS.

Maillard reaction, lipid oxidation, and break down of protein are the fundamental processes that contribute to the production of VOCs in processed foods, thus, enhances flavour development [20,49]. The VOCs’ quantitative and qualitative composition is usually associated with flavour attributes of EWP that subsequently indicates its quality [41]. A total of 65 VOCs were identified and quantified in the control, and the HVCP-treated EWP samples by HS-GC-IMS system as reported in Figure 4 and Table 2. The VOCs were categorised into acids, alcohols, aldehydes, alkanes, cucurbitacins, esters, hydrocarbons, and ketones (Figure 4). For more elaborate observation, the absolute concentrations of the VOCs were plotted on a heatmap as shown in Figure 4. Aldehydes have the highest concentrations (37.41 μg/g) of VOCs, followed by alcohols (29.46 μg/g), hydrocarbons (13.63 μg/g), esters (12.44 μg/g), ketones (3.05 μg/g), alkanes (2.90 μg/g), acids (0.94 μg/g), and cucurbitacins (0.58 μg/g), sequentially. Besides, the VOCs’ concentrations vary significantly among the HVCP treatments. There is no significant difference between the concentrations of 120 and 300 s of HVCP treatments, while there is a significant difference (*p* ≤ 0.05) among the VOCs of control, 60, 120, 180 and 240 s of HVCP treatments (Figure 5b).

#### 2.4.2. Discrimination of Volatile Compounds from Plasma-Treated Egg White Protein

**Figure 6:**
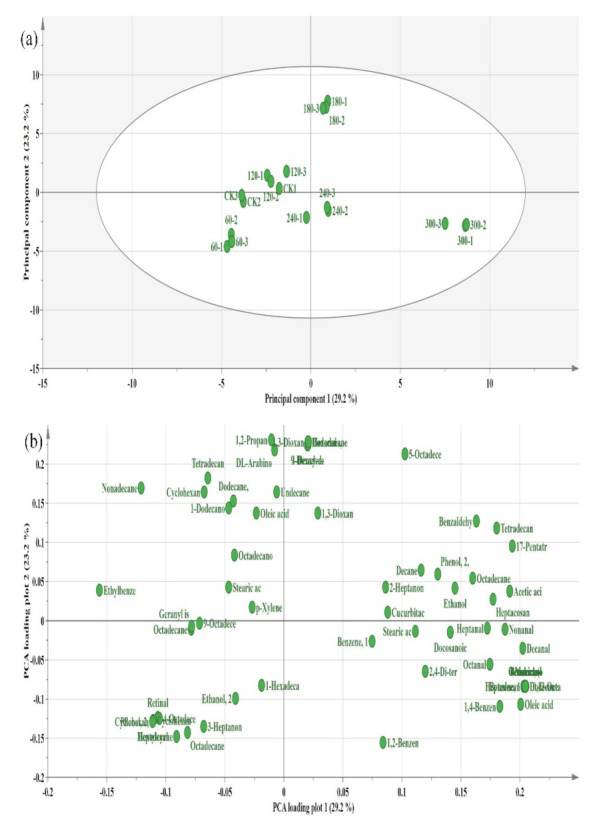
(**a**) PCA plot describing the variation in the volatile compounds’ fingerprints of HVCP-treated EWP; (**b**) Loading plot describing the distribution of volatile compounds’ fingerprints across various HVCP treatment on the EWP. PCA: Principal component analysis.

**Figure 7:**
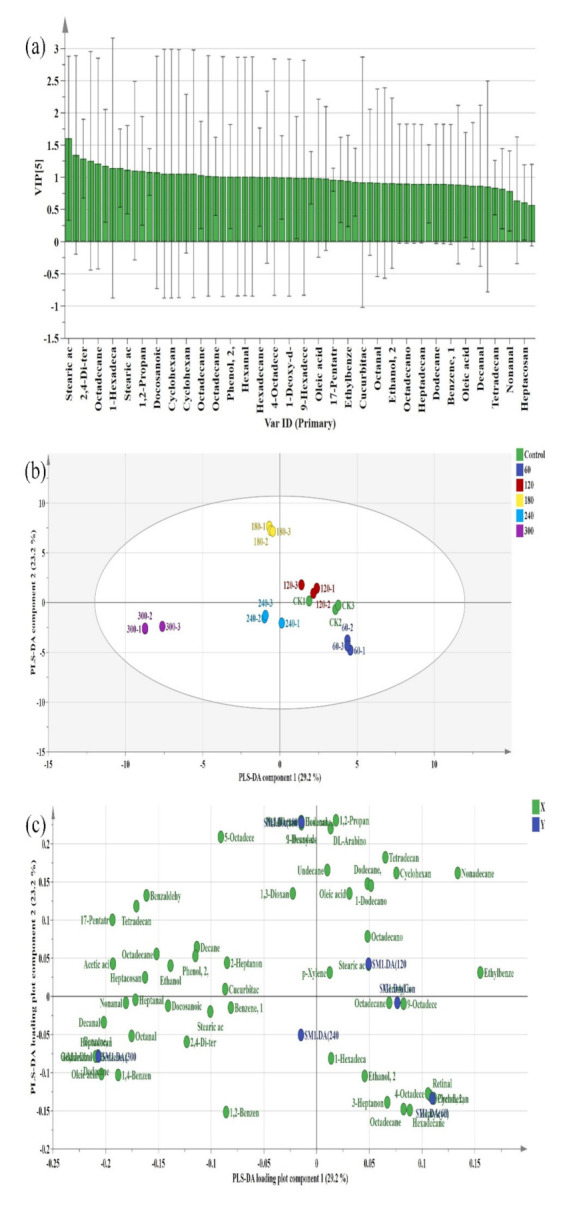
(**a**) The variable importance for the projection (VIP) predictive PLS-DA model of the volatile organic compounds (VOCs); (**b**) PLS-DA score plot showing the variation in the volatile compounds’ fingerprints of HVCP treated EWP; (**c**) PLS-DA loading plot showing the distribution of volatile compounds fingerprints across various HVCP treatment on the EWP. PLS-DA: Partial Least-Square Discriminant Analysis; X: represents the variable; Y: represents the PLS-DA models for the six classes.

Multivariate data analysis (PCA and PLS-DA) was employed to discriminate the HVCP-treated EWP at different treatment times. PCA as an unsupervised data analysis method describes samples based on their classification trends [53]. On the other hand, PLS-DA is a supervised discriminant analysis technique that optimises variables among designated groups which can be used to generate the correlation model between the VOCs and the HVCP treatment times [54]. The variable importance in projection (VIP) scores were used to discriminate the EWP flavour constituents in PLS-DA models with a model sample size of 18 (six treatments × three replications). The HVCP treatment times were taken as Y-variables, while the volatile compounds from the HS-GC-IMS were used as X-variables. The discrimination outcome of the PCA and PLS-DA are shown in Figure 6 and Figure 7, respectively. From the PCA plot, the VOCs were separated clearly based on the HVCP treatments. All the treatments were separated distinctively except for the control and 120 s of HVCP treatment that over-lap each other (Figure 6a). This shows that there is high similarity among the VOCs generated at 120 s and the control. Figure 6b shows the various loadings of VOCs based on their HVCP treatment times.

VIP may be used to examine the weight strength and descriptive capacity of each variable factor on the classification and discrimination of each treatment in the PLS-DA discrimination study [53]. The higher the VIP score (Figure 7a), the greater the difference in the volatile compounds among the HVCP treatments and to the discriminant classification of the flavour of EWP. From Figure 7a, the model segregated the samples noticeably based on the HVCP treatments. This result conforms with the PCA result above as the various HVCP treatments were positioned separately and far from each other (especially among the 60, 180 and 300 s) except for the control and 120 s of HVCP treatment. Conversely, the PLS-DA model’s loading plot displays the various VOCs associated with the models (Figure 7b). This finding corroborated with the PCA loading plot.

#### 2.4.3. Classification of Volatile Compounds from Plasma-Treated Egg White Protein

The VOCs obtained from the HVCP-treated EWP were categorised into various classes. From Figure 5a, the number of VOCs varies with HVCP treatment time but with no definite pattern, 180 s having the highest number of volatiles followed by 300, 60, 240, and 120 s of HVCP treatment consecutively. The control had the lowest number of volatiles, this indicates that HVCP treatment improves the availability of the VOCs. The relationship between VOCs and reactive species is not definite because of the variations in the reactive species’ life span. Thus, the amount and number of VOCs’ generation depend on the life span of the reactive species created by the HVCP [15,55]. Another factor for the variation might be the various intermolecular forces, including non-covalent bonds, van der Waals forces, and hydrogen bonding formed when EWP interact with HVCP [18]. Consequently, this demonstrates the influence of HVCP treatment on the EWP volatile compounds generation, as can be noticed in Table 2.

##### Acids

Eggs contributed to the synthesis of bile acids, subsequently generating Alpha-Muricholic acids [56]. A considerably high concentration (1.16 μg/g) of Alpha-Muricholic acids was generated at 300 s of HVCP treatment only, which might be due to the long exposure of the EWP to the HVCP. The formation of this acid might also be linked to lipid oxidation, as mentioned above.

##### Alcohols

Alcohols were identified in all the treatments at various quantities and concentrations (Figure 5). The HVCP treatments of 120 and 180 s had the equal and highest number of alcohols while 60 s of HVCP treatment had the highest alcohols’ concentration among the control and HVCP-treated samples. The control sample had the least number and concentration of alcohols (Figure 5b). Additionally, 60 and 0 (control) s of HVCP treatments were significantly different (*p* ≤ 0.05) from each other, and other HVCP-treated samples. Furthermore, alcohols were among the VOCs with high concentrations (with a cumulative concentration of 29.46 μg/g) found in the HVCP-treated EWP, ranking second after ketones (Figure 5b). Alcohols are produced owing to lipid oxidation and make an insignificant contribution to food flavour attributes due to their high odour threshold [41]. Harlina, Ma, Shahzad, Gouda and Qiu [49] identified alcohols in clove-extract-augmented duck eggs’ volatile compounds. 

##### Aldehydes

Aldehydes were produced by oxidising polyunsaturated fatty acids (PUFA) and Strecker degradation of amino acids [14,49]. A substantial number of aldehydes were produced, that are 6, 7, 8, 9, 7, and 7 aldehydes for 0 (control), 60, 120. 180, 240 and 300 s of HVCP treatment, respectively (Figure 5a). Among all the samples which are not statistically different, the 120 and 240 s HVCP treatment samples had the highest concentrations of 8.2 and 8.1 μg/g, respectively (Figure 5b). The concentrations of the HVCP-treated samples fluctuated with increased treatment time (Figure 5b). The concentrations of control, 60, 180 and 300 s of HVCP-treated samples were statistically different (*p* ≤ 0.05) from each other. Aldehydes were reported as the major VOCs identified in different breeds of eggs [57].

##### Alkanes

This class of VOCs were found across all the treatment in small numbers (Figure 5b). To be exact, the control group had the highest number and concentration of alkanes, 4 alkanes, and 1.28 μg/g. In addition, the concentration of the control group is significantly different (*p* ≤ 0.05) from the HVCP-treated groups. All the alkanes identified (6-methyl-Octadecane, 2,6,10-trimethyl-Tetradecane, 3-ethyl-5-(2-ethyl butyl)-Octadecane, 1,1-bis(dodecyloxy)-Hexadecane, and (Z,Z)-1,1′-[1,2-ethanediylbis(oxy)]bis-9-Octadecene) are straight-chain alkanes that insignificantly contribute to the flavour of the EWP, which are formed through decarboxylation of fatty acids from glycerides [41]. Xiang, Jin, Gouda, Jin and Ma [57] established similar results for alkanes from different breeds of chicken eggs where hexane, hexadecane, and 2,6,10-trimethyl-Dodecane were the only alkanes identified.

##### Cucurbitacins

Cucurbitacins were only identified across the HVCP-treated groups in minute concentrations. The formation of this compound might be related to the interaction of EWP with ROS, RNS, charged particles, and free radicals produced by HVCP. Table 2 shows that there is no significant difference (*p* ≤ 0.05) among the cucurbitacins’ concentrations across the HVCP treatments. This is the first study that reports cucurbitacins being identified in eggs as per the literature we can lay our hands on. Also, cucurbitacins have shown to possess a strong anticancer activity [58].

##### Esters

Esters are formed due to the esterification reaction between free fatty acids and alcohols [59] or through free radical-induced lipid oxidation due to the HVCP treatment [60]. The number of esters detected across the control and HVCP-treated samples were in large quantities while their concentrations were moderate (Figure 5a,b). The 180 s of HVCP treatment had the highest concentration of esters that are significantly different (*p* ≤ 0.05) from the control and 120 s group; and 60, 240 and 300 s of HVCP treatment group that were not statistically different within-group but different between the two groups.

##### Hydrocarbons

Hydrocarbons were isolated among the main VOCs in duck egg gels [14]. To be precise, these are the most abundant VOCs identified in the HVCP-treated EWP that amount to 20 hydrocarbons. The hydrocarbons identified were mainly aromatic and straight-chain hydrocarbons. The aromatic hydrocarbons are formed from their benzene derivatives. At the same time, straight-chain hydrocarbons might be due to fatty acid decarboxylation from glycerides [20,60]. Despite their abundance, the isolated hydrocarbons had moderate concentrations across the HVCP treatment and control due to their high threshold values [20]. The concentrations of hydrocarbons increase significantly (*p* ≤ 0.05) from 60 to 180 s of HVCP treatment, then decline significantly at 240 s, then increase at 300 s of HVCP treatment.

##### Ketones

Ketones had the lowest concentration of volatile compounds that were identified among the VOCs classes across all the treatments (Table 2). Thus, their contribution to the flavour of the EWP was minute as only two compounds were identified (2-Heptanone and 3-Heptanone). In contrast, ketones were found to contribute significantly to the flavour of salted duck eggs [49], duck egg gels [14], and different breeds of eggs [57]. Ketones are produced from the oxidation of free fatty acids, amino acids decomposition, and free radical-induced lipid oxidation [57,61].

## 3. Materials and Methods

### 3.1. Reagents and Solutions

Deionised water (DIW) (MUL-9000 water purification systems, Nanjing Zongxin Pure Water Equipment Co., Ltd., Nanjing, China). Biuret reagent B3934-110ML, potassium chloride (KCl), silver chloride (AgCl), potassium hydroxide (KOH), ethanol, hydrochloric acid (HCl), n-hexane, sulfosalicylic acid, and tartaric acid were purchased from Sigma-Aldrich, Shanghai, China. N-ketones C4-C9 was provided by Sinopharm Chemical Reagent Beijing Co., Ltd., Beijing, China.

### 3.2. Egg White Protein Preparation

Fresh chicken eggs (medium-sized) were bought from a local supermarket in Xuanwu County, Nanjing, China. The eggs were checked for cracks after they were washed with tap water and cleaned with tissue paper. Cleaned and unbroken eggs were selected for the experiment. The eggs were cracked open, and then the egg white was carefully separated from the egg yolk and chalazae using an egg separator. The egg white was then homogenised in a beaker (sealed with aluminium foil) by a magnetic stirrer (CrystalMS2-P1H, Suzhou Jiemei Electronics Co., Ltd., Suzhou, China) at 4 °C for 2 h. Subsequently, 300 mL of the homogenised egg white was diluted with three times deionised water (DIW) then was gently stirred manually using a glass stirring rod. The egg white protein was quantified as 24.36 mg/mL using the Biuret reagent method at a pH (S20 SevenEasy pH meter, Mettler Toledo, OH, USA) of 8.79. Whereas an electric conductivity meter (INESA DDBJ-350, INESA Scientific Instrument Co., Ltd., Shanghai, China) was used to determine electric conductivity of 2.37 mS/cm, total dissolved solids (TDS) of 1182 mg/L and salinity of 0.14%. The diluted egg white solution was then centrifuged (Allegra 64R Centrifuge, Beckman Coulter, IN, USA) at 11,000× *g* for 30 min at 4 °C to remove impurities and insoluble proteins. 

### 3.3. High-Voltage Cold Plasma Device and Treatment

A high-voltage cold plasma system with a dielectric barrier discharge configuration was used. The device comprised an AC Dielectric Test Set (BK-130, Phenix Technologies, Accident, MD, USA), a high voltage transformer, lower and upper aluminium electrodes of 150 mm diameter each, and high voltage wires upper and lower dielectric barriers made of polypropylene sheets. Twenty mL of EWP was poured into a 90 mm diameter Petri plate, sealed in a Polypropylene (PP) food tray (180 mm × 130 mm × 40 mm, Shanghai Yihao Industrial Co., Ltd., Shanghai, China) under atmospheric air conditions and then treated with HVCP with a treatment gap of 50 mm and a discharge gap 10 mm from the upper electrode. The EWP was treated at 0 (control), 60, 120, 180, 240, and 300 s at a high voltage of 40 kV. The treated EWP was separated into two parts; one portion was lyophilised (Christ Alpha 2-4 LSCplus, Martin Christ Gefriertrocknungsanlagen GmbH, Osterode am Harz, Germany) and stored at −40 °C till further use, while the other was used immediately [62,63,64].

### 3.4. Chemical Components Analysis of Egg White Protein

The moisture, protein, minerals and reducing sugars’ contents of HVCP-treated EWP were determined according to the Official Methods of Analysis of AOAC international [65].

### 3.5. Electronic Nose Analysis of Egg White Protein

AIRSENSE PEN3 (Airsense Analytics GmbH, Schwerin, Germany) E-nose is an intelligent chemical sensor that utilises up to 10 various algorithms based on a metal-oxide gas sensor array to identify the smell of various food materials. According to Qian, et al. [66], it was utilised to investigate the influence of HVCP treatment time on the EWP odour with a slight modification. The E-nose was operated at the chamber and injection flow rates of 200 mL/min, the acquisition time of 90 s, flush time of 60 s after each measurement, and atmospheric air was used as carrier gas. The HVCP-treated EWP of 3 mL was pipetted into 20 mL headspace bottles and were sealed using a 20 mm manual vial crimper. The bottles were held at 37 °C for 20 min to equilibrate the headspace prior to each measurement. A 0.45 μm filter was attached to the E-nose nozzle to prevent sensor surface damage and malfunctioning. Results were acquired using WinMuster PEN evaluation and analysis software (Airsense Analytics GmbH, Schwerin, Germany).

### 3.6. Electronic Tongue Analysis of Egg White Protein

Electronic tongue measurements were conducted using SA402B Insent E-tongue (Intelligent Sensor Technology Inc., Atsugi-Shi, Japan). This taste-sensing system uses an artificial lipid membrane through various sensors that produce electrostatic or hydrophilic interactions with several taste materials, allowing them to sense the taste. Various standard solutions that include internal, reference, negative, and objective solutions were prepared according to the manufacturer’s instructions prior to the experiment. For the internal solution, 248.2 g of potassium chloride (KCl) was dissolved in 1 L of DIW, 10 mg of silver chloride (AgCl) was added, stirred, and heated for at least 8 h. For the reference solution, 2.2365 g of potassium chloride (KCl), and 0.045 g tartaric acid were dissolved in 1 L of DIW. For the negative solution, 300 mL ethanol and 8.3 mL of hydrochloric acid (HCl) were added to 500 mL of DIW, and the volume was made up to 1 L with DIW. Lastly, the objective solution was made by adding 7.46 g of KCl to 500 mL of DIW, then 300 mL of ethanol and 0.56 g of potassium hydroxide (KOH) were added, and the volume was made up to 1 L of DIW. 400 μL of the internal solution was added to the sensors and immersed 3.33 M of KCl for at least 24 h prior to the experiment to condition the electrodes. The taste sensors analyse the taste by measuring the difference between the potential of a reference solution (V_r_) and the potential of the samples (V_s_) as below:

$$V_{s}-{\;V}_{r}=\mathrm{Relative}\;\mathrm{taste}\;\mathrm{value}$$

The E-tongue measurements were evaluated using a method by Zhang, et al. [67] with a slight alteration. 20 mL of HVCP-treated EWP sample was dissolved in 100 mL of DIW and was vortexed (Crystal VM-01U, Suzhou Jiemei Electronics Co., Ltd., Suzhou, China) for 2 min. Then the solution was centrifuged (Allegra 64R Centrifuge, Beckman Coulter, IN, USA) at 2000× *g* for 15 min at 4 °C. Before E-tongue measurements, the supernatant was filtered to eliminate insoluble materials using Whatman No. 1 (11 μm) filter paper.

### 3.7. Headspace-Gas Chromatography-Ion Mobility Spectrometry

An Agilent 490 gas chromatograph (Agilent Technologies, Palo Alto, CA, USA) and IMS device (FlavorSpec^®^, Gesellschaft für Analytische Sensorsysteme mbH, Dortmund, Germany) with an automatic sampling unit (CTC Analytics AG, Zwingen, Switzerland) attached were utilised to determine the volatile compounds of the HVCP-treated EWP samples based on Luo, Nasiru, Zhuang, Zhou and Zhang [59]. Then 0.5 g of lyophilised EWP powder from each treatment was transferred into 20 mL headspace bottles. The automatic sampling unit was operated under the following conditions: incubation volume of 500 μL, syringe temperature of 85 °C, incubation temperature of 60 °C, incubation time of 20 min, and incubation speed of 500 rpm. The GC was performed at 45 °C with a fused silica capillary column (FS-SE-54-CB 15 m 0.53 mm ID) to identify the volatile components. Nitrogen (99.99% purity) was utilised as the carrier gas, with a programmed flow ramp of 2 mL/min for 2 min, 15 mL/min for 10 min, 100 mL/min for 20 min, 150 mL/min at 30 min, and then the flow ceased. The samples were separated in a column at 60 °C and then ionised for 30 min in an IMS ionisation chamber at 45 °C. The drift gas (nitrogen gas) was set at 150 mL/min. The volatile compounds’ retention index (RI) was calculated using N-ketones C4-C9 (Sinopharm Chemical Reagent Beijing Co., Ltd., Beijing, China) as external references. The RI and drift time (the time it takes for ions to reach the collector through a drift tube, in milliseconds) of standards in the GC-IMS library (Gesellschaft für Analytische Sensorsysteme mbH, Dortmund, Germany) were compared to identify volatile compounds. GC-IMS library search, instrumental laboratory analytical viewer (LAV, Gesellschaft für Analytische Sensorsysteme mbH, Dortmund, Germany), and three plug-ins were used to analyse the spectra (Gesellschaft für Analytische Sensorsysteme mbH, Dortmund, Germany). The VOCs were identified by comparing the mass spectra in the NIST 20 and Wiley Libraries (NIST & Wiley 7.0, John Wiley & Sons, Inc., Hoboken, NJ, USA), and the results were expressed in areas units (×10^6^). 

### 3.8. Statistical Analysis

Statistical analysis, principal component analysis (PCA) for E-nose and E-tongue, radar plots and heatmap were generated using Origin graphing and statistical analysis software (version 2021b, OriginLab Corporation, Northamptom, MA, USA). Statistical differences were evaluated using a one-way analysis of variance (ANOVA) under Fisher’s Least Significant Difference (LSD) Test. The statistical significance level was set as 0.05. Five and four replications were used for PCA analysis for E-nose and E-tongue, respectively. For the volatile compounds result, unsupervised PCA and supervised partial least square-discriminant analysis (PLS-DA) were employed using SIMCA 14.1 (Umetrics, Umea, Sweden) to discriminate the contribution of each volatile compound of the EWP at different HVCP treatment times. All experiments were performed three times except for E-tongue, where it was performed four times.

## 4. Conclusions

In a nutshell, high-voltage cold-plasma treatment could significantly enhance the flavour attributes of egg white protein. Principal component analysis of E-nose data clearly discriminates the HVCP treated samples having a good response from W1S, W2W, W1C, W3C, and W5C odour sensors with favourable results at 60 and 120 s of HVCP treatment. E-tongue results correspond to the E-nose results, which show 60 and 120 s of HVCP treated samples were distinctively separated on PCA plot and had a better response from taste sensors than other treatments. The qualitative and quantitative analysis of EWP volatiles compounds using HS-GC-IMS identified 65 compounds across the control and HVCP treated samples, illustrating an overall increase in the concentrations of the volatile compounds as the HVCP treatment time increases. Equally, butyl-cyclohexane, heptylcyclohexane, 2,6,10-trimethyl-dodecane and 2,6-bis(1,1-dimethylethyl)-phenol are the major VOCs that distinctively discriminate HVCP treatment of 60 s, while 1,2- propanediol for 120 s, acetic acid, hexanal, 1,2-dibromo-dodecane and hexadecane for 180 s, 3-heptanone, oleic acid, and 2,6-bis(1,1-dimethylethyl)-1,4-Benzenediol for 240 s and octadecanoic acid, heptadecane, Alpha-Muricholic acids, decanal and (1-methylethyl)-Benzene for 300 s of HVCP treatments, respectively, give each treatment its distinctive VOCs fingerprints. Moreover, the fusion of E-nose, E-tongue, and HS-GC-IMS could clearly discriminate the flavour attributes of EWP at different HVCP treatment times. In the future, different HVCP treatment voltage, treatment frequency, and other treatment conditions such as exposure mode and treatment gas could be investigated to have more information on the effect of HVCP on EWP for food industries to utilise this novel technology.

## Figures and Tables

**Table 1:** Chemical components composition of HVCP treated EWP.

Chemical Component Composition (%*w*/*w*)	Treatment Time (s)					
	0	60	120	180	240	300
**Moisture content**	88.33 ± 0.58 ^a^	88.33 ± 0.58 ^a^	88.37 ± 1.00 ^a^	87.77 ± 0.55 ^a^	87.43 ± 0.78 ^a^	88.33 ± 0.79 ^a^
**Protein**	11.00 ± 0.00 ^a^	10.83 ± 0.21 ^a^	10.83 ± 0.21 ^a^	10.79 ± 0.17 ^a^	10.92 ± 0.07 ^a^	11.00 ± 0.07 ^a^
**Minerals**	0.79 ± 0.01 ^a^	0.79 ± 0.01 ^a^	0.75 ± 0.06 ^a^	0.80 ± 0.13 ^a^	0.95 ± 0.03 ^b^	0.95 ± 0.03 ^b^
**Reducing sugars**	0.19 ± 0.01 ^a^	0.18 ± 0.02 ^a^	0.18 ± 0.02 ^a^	0.18 ± 0.02 ^a^	0.19 ± 0.01 ^a^	0.19 ± 0.01 ^a^

Data = mean ± SD. SD: Standard deviation. Different superscript letters (a, b) mean values are significantly different (*p* ≤ 0.05) across the treatment time. %*w*/*w*: percentage weight per weight.

**Table 2:** Volatile compounds identified in the egg white protein-treated high-voltage cold plasma using HS-GC-IMS.

Classification	RT	Volatile Compounds Attributes					Area (%)					
		Compound Name	SI	RSI	CAS No.	Formula	Control	60 s	120 s	180 s	240 s	300 s
Acids	43.03	Alpha-Muricholic acid	607	629	2393-58-0	C_24_H_40_O_5_	-	-	-	-	-	1.48 ± 0.11 ^vA^
	**Total**											**1.48 ± 0.11**
Alcohols	3.08	Ethanol	860	867	64-17-5	C_2_H_6_O	0.39 ± 0.04 ^aA^	0.41 ± 0.06 ^aA^	0.50 ± 0.09 ^aB^	0.58 ± 0.12 ^aB^	0.45 ± 0.36 ^aB^	0.65 ± 0.15 ^aC^
	4.82	1-Deoxy-d-mannitol	639	676	60965-81-3	C_14_H_14_O_5_	-	-	-	0.02 ± 0.01 ^bA^	-	-
	21.42	1,4-Benzenediol, 2,6-bis(1,1-dimethylethyl)-	608	629	2444-28-2	C_14_H_22_O_2_	-	-	-	-	0.19 ± 0.03 ^cA^	0.19 ± 0.02 ^iB^
	33.31	Ethanol, 2-(octadecyloxy)-	727	755	2136-72-3	C_20_H_42_O_2_	-	0.22 ± 0.03 ^mA^	0.23 ± 0.08 ^iA^	0.26 ± 0.08 ^oB^	0.30 ± 0.27 ^aB^	0.37 ± 0.02 ^oB^
	34.46	1-Hexadecanol, 2-methyl-	694	720	2490-48-4	C_17_H_36_O	-	0.18 ± 0.03 ^hA^	-	-	0.66 ± 0.31 ^wB^	-
	34.64	1-Dodecanol, 3,7,11-trimethyl-	725	755	6750-34-1	C_15_H_32_O	-	0.18 ± 0.10 ^hA^	0.25 ± 0.11 ^dA^	0.66 ± 0.31 ^pB^	-	-
	36.94	Phenol, 2,6-bis(1,1-dimethylethyl)-	875	927	128-39-2	C_14_H_22_O	-	11.01 ± 1.11 ^oA^	-	-	-	-
	36.94	2,4-Di-tert-butylphenol	889	889	96-76-4	C_14_H_22_O	-	-	8.98 ± 0.64 ^oA^	-	7.82 ± 0.58 ^lA^	6.86 ± 0.82 ^qA^
	39.15	Phenol, 2,6-bis(1,1-dimethylethyl)-4-(1-methylpropyl)-	815	857	17540-75-9	C_18_H_30_O	0.59 ± 0.10 ^hA^	0.52 ± 0.02 ^pB^	0.53 ± 0.06 ^pC^	0.63 ± 0.03 ^yD^	0.54 ± 0.06 ^mE^	0.61 ± 0.02 ^tF^
	**Total**						**0.98 ± 0.14 ^A^**	**12.52 ± 1.33 ^B^**	**10.49 ± 0.98 ^C^**	**2.15 ± 0.55 ^D^**	**9.96 ± 1.61 ^E^**	**10.29 ± 1.03 ^F^**
Aldehydes	4.81	DL-Arabinose	628	662	20235-19-2	C_5_H_10_O_5_	-	-	0.01 ± 0.01 ^bA^	0.02 ± 0.01 ^bA^	-	0.01 ± 0.01 ^bA^
	12.63	Hexanal	709	893	66-25-1	C_6_H_12_O	-	-	-	0.19 ± 0.02 ^dA^	-	-
	17.59	Heptanal	717	810	111-71-7	C_7_H_14_O	0.09 ± 0.01 ^aA^	0.14 ± 0.03 ^dB^	0.16 ± 0.02 ^iC^	0.18 ± 0.02 ^dB^	0.23 ± 0.03 ^cE^	0.20 ± 0.02 ^fE^
	21.12	Cyclohexanone, 2-ethyl-	756	779	4423-94-3	C_8_H_14_O	5.07 ± 1.31 ^eA^	-	6.6 ± 0.30 ^kA^	5.39 ± 0.65 ^jA^	5.84 ± 0.21 ^gA^	-
	21.77	Octanal	748	889	124-13-0	C_8_H_16_O	0.10 ± 0.1 ^aA^	0.14 ± 0.02 ^dB^	0.14 ± 0.03 ^iC^	0.15 ± 0.02 ^eB^	0.21 ± 0.02 ^cD^	0.19 ± 0.01 ^iD^
	25.43	Nonanal	818	876	124-19-6	C_9_H_18_O	2.90 ± 0.38 ^fA^	3.68 ± 0.34 ^ìA^	4.03 ± 0.36 ^mA^	3.83 ± 0.30 ^kB^	4.77 ± 0.11 ^hC^	4.52 ± 0.35 ^lD^
	20.28	Benzaldehyde	636	873	100-52-7	C_7_H_6_O	-	-	0.59 ± 0.03 ^jA^	0.61 ± 0.03 ^iB^	0.44 ± 0.12 ^aB^	0.55 ± 0.05 ^hB^
	27.26	Retinal	603	704	116-31-4	C_20_H_28_O	-	0.13 ± 0.03 ^dA^	-	-	-	-
	28.74	Decanal	838	873	112-31-4	C_20_H_28_O	0.54 ± 0.05 ^aA^	0.72 ± 0.05 ^jA^	0.7 ± 0.17 ^nA^	0.87 ± 0.05 ^mB^	1.09 ± 0.05 ^iC^	1.15 ± 0.15 ^nD^
	34.09	4-Octadecenal	716	747	56554-98-4	C_18_H_34_O	-	0.11 ± 0.04 ^gA^	-	-	-	-
	38.32	5-Octadecenal	724	757	56554-88-2	C_18_H_34_O	0.30 ± 0.10 ^aA^	0.20 ± 0.13 ^hB^	0.49 ± 0.13 ^aC^	0.82 ± 0.08 ^vD^	0.33 ± 0.06 ^aA^	0.46 ± 0.03 ^rC^
	**Total**						**9.00 ± 1.95 ^A^**	**5.12 ± 0.64 ^B^**	**12.72 ± 1.18 ^C^**	**12.06 ± 1.15 ^D^**	**12.92 ± 0.60 ^E^**	**7.08 ± 0.62 ^F^**
Alkanes	28.82	Octadecane, 6-methyl-	705	785	10544-96-4	C_19_H_40_	0.23 ± 0.14 ^aA^	-	-	-	-	-
	33.45	Hexadecane, 1,1-bis(dodecyloxy)-	727	733	56554-64-4	C_40_H_82_O_2_	-	0.20 ± 0.04 ^hA^	-	-	0.14 ± 0.05 ^cB^	-
	36.75	Tetradecane, 2,6,10-trimethyl-	739	815	14905-56-7	C_17_H_36_	0.42 ± 0.15 ^aA^	-	0.36 ± 0.24 ^eB^	0.46 ± 0.27 ^sA^	-	0.55 ± 0.27 ^hA^
	41.04	(Z,Z)-9-Octadecene, 1,1’-[1,2-ethanediylbis(oxy)]bis-	678	695	17367-13-4	C_38_H_74_O_2_	0.22 ± 0.12 ^aA^	-	0.18 ± 0.02 ^iA^	-	0.16 ± 0.02 ^cA^	-
	41.59	Octadecane, 3-ethyl-5-(2-ethylbutyl)-	715	721	55282-12-7	C_26_H_54_	0.41 ± 0.23 ^aA^	-	-	0.51 ± 0.02 ^aB^	-	0.72 ± 0.21 ^uC^
	**Total**						**1.28 ± 0.64 ^A^**	**0.20 ± 0.04 ^B^**	**0.54 ± 0.26 ^C^**	**0.97 ± 0.29 ^D^**	**0.30 ± 0.07 ^E^**	**1.27 ± 0.48 ^F^**
Cucurbitacins	36.28	Cucurbitacin B, dihydro-	600	661	13201-14-4	C_32_H_48_O_8_	-	0.18 ± 0.03 ^hA^	0.13 ± 0.07 ^iA^	0.20 ± 0.04 ^dA^	0.22 ± 0.03 ^cA^	0.18 ± 0.10 ^jA^
	**Total**							**0.18 ± 0.03 ^A^**	**0.13 ± 0.07 ^A^**	**0.20 ± 0.04 ^A^**	**0.22 ± 0.03 ^A^**	**0.18 ± 0.10 ^A^**
Esters	9.05	Acetic acid, hydroxy-, ethyl ester	867	970	623-50-7	C_4_H_8_O_3_	1.18 ± 0.14 ^bA^	1.25 ± 0.09 ^bA^	1.47 ± 0.16 ^cA^	1.79 ± 0.14 ^cB^	1.89 ± 0.25 ^bB^	1.91 ± 0.34 ^cB^
	24.60	10,13-Octadecadiynoic acid, methyl ester	615	621	18202-24-9	C_19_H_30_O_2_	-	-	-	-	-	0.01 ± 0.00 ^bA^
	25.80	(Z)-Stearic acid, 2-(9-octadecenyloxy)ethyl ester	627	648	29027-97-2	C_38_H_74_O_3_	-	-	0.04 ± 0.01 ^fA^	-	-	-
	29.09	1,3-Dioxane, 4-(hexadecyloxy)-2-pentadecyl-	603	725	34315-34-9	C_35_H_70_O_3_	-	-	-	0.22 ± 0.06 ^dA^	0.23 ± 0.06 ^cA^	-
	31.03	Stearic acid, 3-(octadecyloxy)propyl ester	553	642	17367-40-7	C_39_H_78_O_3_	0.33 ± 0.22 ^aA^	0.39 ± 0.20 ^lA^	-	0.42 ± 0.08 ^nA^	0.27 ± 0.11 ^dB^	0.5 ± 0.03 ^mC^
	31.15	Octadecanoic acid, 2-(octadecyloxy)ethyl ester	644	658	28843-25-6	C_38_H_76_O_3_	-	-	-	-	-	0.26 ± 0.02 ^eA^
	33.19	1,2-Propanediol, 3-(octadecyloxy)-, diacetate	631	679	21994-81-0	C_25_H_48_O_5_	-	-	0.16 ± 0.06 ^iA^	0.20 ± 0.03 ^dB^	-	-
	33.57	Octadecane, 1,1’-[(1-methyl-1,2-ethanediyl)bis(oxy)]bis-	562	673	35545-51-8	C_39_H_80_O_2_	-	0.56 ± 0.04 ^kA^	-	-	0.47 ± 0.12 ^aA^	-
	36.09	Geranyl isovalerate	625	660	109-20-6	C_15_H_26_O_2_	0.09 ± 0.03 ^aA^	-	-	-	-	-
	37.31	Oleic acid, 3-(octadecyloxy)propyl ester	678	709	17367-41-8	C_39_H_76_O_3_	-	0.13 ± 0.13 ^dA^	0.13 ± 0.09 ^iB^	0.33 ± 0.04 ^uC^	0.29 ± 0.04 ^aC^	-
	39.55	Docosanoic acid, 8,9-dihydroxy-, methyl ester	616	635	56555-06-7	C_23_H_46_O_4_	-	0.43 ± 0.04 ^qA^	-	0.52 ± 0.03 ^zB^	0.53 ± 0.03 ^mB^	0.55 ± 0.03 ^hC^
	39.99	Octadecanoic acid, 4-hydroxy-, methyl ester	627	681	2420-38-4	C_19_H_76_O_4_	-	0.22 ± 0.15 ^hA^	-	0.27 ± 0.13 ^tB^	-	-
	41.05	(Z,Z)-9-Hexadecenoic acid, 9-octadecenyl ester	672	691	22393-98-2	C_34_H_64_O_2_	-	-	-	0.22 ± 0.04 ^cA^	-	-
	41.50	Oleic acid, eicosyl ester	655	670	22393-88-0	C_38_H_74_O_2_	-	-	-	-	0.14 ± 0.02 ^cA^	0.20 ± 0.04 ^fB^
	43.02	1,3-Dioxane, 5-(hexadecyloxy)-2-pentadecyl-, trans-	603	725	56599-40-7	C_35_H_70_O_3_	-	-	-	1.5 ± 0.04 ^cA^	-	-
	43.22	1,2-Benzenedicarboxylic acid, butyl octyl ester	741	803	84-78-6	C_20_H_30_O_4_	-	0.49 ± 0.21 ^rA^	-	-	-	0.5 ± 0.16 ^mB^
	**Total**						**1.60 ± 0.39 ^A^**	**3.47 ± 0.86 ^B^**	**1.80 ± 0.32 ^C^**	**5.47 ± 0.55 ^D^**	**3.82 ± 0.63 ^E^**	**3.43 ± 0.58 ^F^**
Hydrocarbons	16.05	Ethylbenzene	737	887	100-41-4	C_8_H_10_	0.16 ± 0.12 ^aA^	0.24 ± 0.01 ^cA^	0.24 ± 0.13 ^dA^	0.14 ± 0.09 ^eA^	0.17 ± 0.11 ^cA^	0.09 ± 0.00 ^dA^
	16.08	Benzene, 1,3-dimethyl-	843	873	108-38-3	C_8_H_10_	-	0.14 ± 0.10 ^dA^	0.25 ± 0.16 ^dB^	0.15 ± 0.11 ^eA^	0.28 ± 0.17 ^dB^	0.21 ± 0.10 ^fB^
	16.29	p-Xylene	831	888	106-42-3	C_8_H_10_	0.37 ± 0.21 ^aA^	0.24 ± 0.01 ^cA^	0.34 ± 0.07 ^eA^	0.29 ± 0.05 ^fA^	0.34 ± 0.08 ^aA^	0.26 ± 0.02 ^eA^
	18.69	Benzene, (1-methylethyl)-	661	772	98-82-8	C_9_H_12_	-	-	-	-	-	0.04 ± 0.01 ^aD^
	21.68	Dodecane, 1,2-dibromo-	606	675	55334-42-4	C_12_H_24_Br_2_	-	-	-	0.14 ± 0.01 ^eA^	-	-
	21.96	Decane	622	871	124-18-5	C_10_H_22_	0.1 ± 0.04 ^aA^	-	-	-	-	-
	23.12	Cyclohexane, butyl-	740	812	4292-92-6	C_11_H_22_	-	0.10 ± 0.01 ^gA^	-	-	-	-
	23.93	Heptylcyclohexane	694	781	5617-41-4	C_13_H_26_	-	0.12 ± 0.02 ^dA^	-	-	0.12 ± 0.03 ^cA^	0.16 ± 0.02 ^jB^
	24.77	Dodecane, 2,6,11-trimethyl-	717	864	31295-56-4	C_15_H_32_	-	-	-	-	-	0.31 ± 0.09 ^bA^
	24.95	Dodecane, 2,6,10-trimethyl-	773	859	3891-98-3	C_15_H_32_	-	0.18 ± 0.07 ^hA^	-	0.34 ± 0.19 ^lB^	-	-
	25.29	Undecane	713	869	1120-21-4	C_11_H_24_	-	-	0.29 ± 0.01 ^lA^	0.30 ± 0.02 ^fB^	0.32 ± 0.02 ^aB^	-
	26.73	Cyclohexane, pentyl-	680	821	4292-92-6	C_11_H_22_	-	0.12 ± 0.02 ^dA^	-	-	-	-
	28.55	Dodecane	725	799	112-40-3	C_12_H_44_	-	-	-	-	-	0.50 ± 0.03 ^mA^
	31.60	Heptadecane, 2,6,10,15-tetramethyl-	717	815	54833-48-6	C_21_H_44_	-	-	-	-	-	0.28 ± 0.03 ^eA^
	34.36	Tetradecane	802	904	629-59-4	C_10_H_30_	0.66 ± 0.26 ^gA^	-	0.64 ± 0.22 ^nB^	1.00 ± 0.12 ^rC^	0.71 ± 0.19 ^jB^	1.04 ± 0.01 ^pD^
	35.79	Nonadecane	804	904	629-92-5	C_19_H_40_	0.66 ± 0.25 ^gA^	0.59 ± 0.02 ^kA^	0.71 ± 0.16 ^nA^	0.96 ± 0.08 ^xB^	-	-
	38.36	Heptacosane	783	823	593-49-7	C_27_H_56_	0.54 ± 0.15 ^aA^	0.55 ± 0.03 ^kA^	0.67 ± 0.09 ^nA^	0.73 ± 0.22 ^qB^	0.66 ± 0.05 ^kA^	0.84 ± 0.15 ^sC^
	38.64	Hexadecane	799	874	544-76-3	C_16_H_34_	-	-	-	0.95 ± 0.07 ^xA^	-	-
	41.49	Benzene, 1,1’-(1,2-cyclobutanediyl)bis-, trans-	678	868	20071-09-4	C_16_H_16_	-	-	-	-	-	0.71 ± 0.09 ^aA^
	41.72	17-Pentatriacontene	658	672	6971-40-0	C_35_H_70_	-	-	0.32 ± 0.04 ^qA^	0.41 ± 0.01 ^nB^	0.33 ± 0.03 ^aA^	0.45 ± 0.01 ^wC^
	**Total**						**2.49 ± 1.03 ^A^**	**2.28 ± 0.29 ^B^**	**3.46 ± 0.89 ^C^**	**5.41 ± 0.97 ^D^**	**2.93 ± 0.68 ^E^**	**4.89 ± 0.55 ^F^**
Ketones	17.01	2-Heptanone	660	744	110-43-0	C_7_H_14_O	0.05 ± 0.01 ^cA^	0.06 ± 0.01 ^eB^	0.07 ± 0.00 ^gC^	0.07 ± 0.01 ^gD^	-	0.09 ± 0.01 ^dE^
	16.88	3-Heptanone	652	730	106-35-4	C_7_H_14_O	-	0.06 ± 0.01 ^eA^	-	-	0.08 ± 0.02	-
	**Total**						**0.05± 0.01 ^A^**	**0.12 ± 0.02 ^B^**	**0.07 ± 0.00 ^C^**	**0.07 ± 0.01 ^C^**	**0.08 ± 0.02 ^D^**	**0.09 ± 0.01 ^E^**

RT: Retention time; SI: Match factor; RSI: Reverse match factor. Lower case letters (a–z) means values are significantly different (*p* ≤ 0.05) across the treatment time. Upper case letters (A–F) means values are significantly different (*p* ≤ 0.05) across the volatile compounds’ area (area unit × 10^6^). Values are mean ± standard deviation, *n =* 3.

## Data Availability

Not applicable.
